# UK multicentre real-world data of the use of cyclin-dependent kinase 4/6 inhibitors in metastatic breast cancer

**DOI:** 10.1016/j.esmorw.2024.100064

**Published:** 2024-08-20

**Authors:** G. Gullick, C.N. Owen, W.J. Watkins, S. Cook, J. Helbrow, H. Reed, R. Squires, S. Park, E. Weir, F. Aquilina, N. Webber, E. Nye, C. Atkinson, C. Blair, A. Halstead, E. Daniels, A. Alves, S. Chew, W. Thomas, S. Spensley, M. Beresford, R. Bowen, T. Robinson

**Affiliations:** 1 Bristol Cancer Institute, University Hospitals Bristol and Weston NHS Foundation Trust, Bristol; 2 Royal United Hospitals Bath NHS Foundation Trust, Bath; 3 Department of Infection and Immunity, School of Medicine, University of Cardiff, Cardiff; 4 Somerset NHS Foundation Trust, Taunton; 5 Gloucestershire Hospitals NHS Foundation Trust, Gloucester; 6 Royal Devon University Healthcare NHS Foundation Trust, Exeter; 7 Population Health Sciences, Bristol Medical School, University of Bristol, Bristol, UK

**Keywords:** HR+/HER2− locally advanced and metastatic breast cancer, cyclin-dependent kinase 4/6 inhibitors (CDK4/6i), real-world evidence, efficacy, survival data, toxicity

## Abstract

**Background:**

Cyclin-dependent kinase 4/6 inhibitors (CDK4/6is) are widely used to treat hormone receptor-positive (HR+)/ human epidermal growth factor receptor 2-negative (HER2−) metastatic breast cancer (MBC). This study aimed to capture the real-world efficacy and tolerability of CDK 4/6is.

**Patients and methods:**

Data were retrospectively collected from five centres in South West England between April 2017 and November 2022.

**Results:**

Six hundred and sixty-six patients were included (median age 66 years; interquartile range 23-92 years). Five hundred and forty-four (82.7%) were treated with CDK4/6i as first-line therapy and 122 (18.3%) as second-line therapy. Median follow-up time was 28 months (range 0-76 months). Five hundred and thirty-seven received palbociclib (80.6%), 85 patients received abemaciclib (12.8%) and 44 received ribociclib (6.6%). Palbociclib and ribociclib most frequently caused neutropenia (38.2% and 26.4%, respectively) whilst abemaciclib caused diarrhoea (61.2%). Rates of dose reduction (DR) (between 53.8% and 59.2%) and time to first DR were similar for all agents (2-3 cycles). For first-line therapy, median progression-free survival (PFS) was 31 months (25-35 months) for palbociclib, 16 months [9 months-not reached (NR)] for abemaciclib and 44 months (21-NR) for ribociclib. Median overall survival (OS) was 47 months (41 months-NR) for palbociclib and was not reached for abemaciclib or ribociclib. Low patient numbers precluded analysis of second-line therapy. On multivariate analysis, visceral metastases and Eastern Cooperative Oncology Group performance status were associated with shorter PFS and OS, whilst DR was associated with longer PFS and OS.

**Conclusion:**

These data demonstrate that CDK4/6is are an effective and safe treatment for metastatic HR+/HER2− breast cancer. Efficacy was in line with trial data and other real-world data. DR was associated with improved PFS and OS, suggesting that trials of CDK4/6is at a lower starting dose are warranted.

## Highlights

• CDK4/6is are an effective and safe treatment for metastatic HR+/HER2− MBC.
• rates of dose reduction appeared higher than in trials although they did not negatively impact PFS/OS.
• The side-effect profile for all three agents mirrored those reported in randomised control trial data.
• Age, CDK4/6i agent, ET backbone, site of metastatic disease and performance status appeared to impact PFS and OS.

## Introduction

Approximately 2.3 million women are diagnosed with breast cancer annually and it is the leading cause of female cancer-related mortality.^1^ Breast cancer is subclassified by both hormone receptor (HR) and human epidermal growth factor receptor 2 (HER2) status, with HR-negative and HER2-negative (HER2−) cancers categorised as triple-negative breast cancer. The majority, 71%, are HR-positive (HR+) and HER2−.^2^ The recommended first-line treatment for HR+/HER2− locally advanced and metastatic breast cancer (MBC) are cyclin-dependent kinase 4/6 inhibitors (CDK4/6is) in combination with endocrine therapy (ET).^3^^,^^4^ CDK4/6is selectively and reversibly inhibit CDK4 and 6. They block cell progression through the cell cycle from G1 to S phase by inactivating the retinoblastoma (Rb) protein,^4^ thereby preventing breast cancer cell proliferation.^5^

Currently, three CDK4/6is are licensed for the treatment of breast cancer: palbociclib, ribociclib and abemaciclib. Varying efficacy and side-effect profiles have been demonstrated in registrational clinical trials (Table 1)^6^, ^7^, ^8^ and in previous real-world data (RWD) studies (Supplementary Table S1, available at https://doi.org/10.1016/j.esmorw.2024.100064). RWD have found efficacy and safety of CDK4/6is comparable to clinical trial data,^9^, ^10^, ^11^ though many patients (14%-57% in RWD, 30%-60% in trials) require a dose reduction (DR) due to toxicity. In multiple RWD analyses, DRs do not appear to compromise efficacy^10^ and in fact have been associated with longer progression-free survival (PFS).^11^^,^^12^ Older patients experience higher rates of toxicity and DR.^10^^,^^11^ Data on efficacy in older patients are conflicting, with some studies reporting shorter PFS^10^^,^^13^ and overall survival (OS)^13^ with increasing age, whereas others report no association^11^ or a longer PFS with increasing age.^14^ Conflicting results could result from non-uniform approaches to age stratification across studies.

**Table 1:** A summary of relevant RCTs for CDK4/6i detailing the name of RCT, trial design and setting, patient selection, median PFS and OS, top three most common any-grade toxicities, dose reduction, median time to first dose reduction and discontinuation rates

Name of RCT	Trial design experimental/comparator arms	Setting	Patient selection	Median PFS (months)	Median OS (months)	Most common any-grade toxicities in the treatment group	Rates of dose reduction	Median time to first dose reduction	Discontinuation rates
PALOMA-^30^^,^^31^	Phase II RCT Palbociclib + letrozole/letrozole	First line MBC	HR+/HER2− postmenopausal	24.8 (95% CI 22.1-not estimable) versus 14.5 (95% CI 12.9-17.1) HR 0.58 *P* = <0.001	53.9 (95% CI 49.8-60.8) versus 51.2 (95% CI 43.7 -58.9) HR 0.956 (95% CI 0.777-1.177) *P* = 0.34	1. Neutropenia (79.5%) 2. Leukopenia (39.0%) 3. Fatigue (37.4%)	36.0%^31^	3.0 months	11.1%^32^
PALOMA-3^33^^,^^34^	Phase III RCT Palbociclib + fulvestrant/Placebo + fulvestrant	Progressed on previous ET	HR+/HER2− postmenopausal or pre/perimenopausal with GnRHa	9.5 (95% CI 9.2-11.0) versus 4.6 (95% CI 3.5-5.6) HR 0.46 *P* = <0.0001	34.8 (95% CI 28.8-39.9) versus 28.0 (95% CI 23.5-33.8) HR 0.81 (95% CI 0.644-1.029) *P* = 0.09	1. Neutropenia (84.1%) 2. Leukopenia (60.0%) 3. Fatigue (44.1%)	34.0%^34^	70 days^35^	
MONALEESA-2^7^^,^^19^^,^^36^	Phase III RCT Ribociclib + letrozole/letrozole	First line MBC	HR+/HER2− postmenopausal Recurrent/metastatic No prior ET ECOG PS 0/1	25.3 (95% CI 23.0-30.3) versus 16.0 (95% CI 13.4-18.2) HR 0.56 *P* = 9.63 × 10^−^^8^	63.9 (95% CI, 52.4 to 71.0) versus 51.4 (95% CI, 47.2 to 59.7) HR 0.76 (95% CI 0.63-0.93) *P* = 0.008	1. Neutropenia (74.3%) 2. Nausea (51.5%) 3. Fatigue (36.5%)	57.5%^7^	3.0 months	14.6%^23^
MONALEESA-3^20^^,^^37^	Phase III RCT Ribociclib + fulvestrant/Placebo + fulvestrant	≤First line of prior ET	HR+/HER2− postmenopausal or men Locally recurrent or metastatic ECOG PS 0/1 No prior treatment with fulvestrant No prior chemotherapy for advanced disease	20.5 (95% CI 18.5-23.5) versus 12.8 (95% CI 10.9-16.3) HR 0.593 *P* = <0.001	67.7 (95% CI 59.6-not estimable) versus 51.8 (95% CI 40.4-61.2) HR 0.75 (95% CI 0.58-0.97) *P* = 0.03	1. Neutropenia (71.6%) 2. Infections (57.8%) 3. Pulmonary disorders (37.3%)	38.7%^38^	2.8 months	
MONALEESA-7^39^^,^^40^	Phase III RCT Ribociclib + tamoxifen or AI/Placebo + tamoxifen or AI	First line metastatic or in the metastatic setting prior chemotherapy allowed	HR+/HER2− premenopausal ECOG PS 0/1	23.8 (95% CI 19.2-not reached) versus 13 (95% CI 11.0-16.4) HR 0.55 *P* = <0.0001	58.7 versus 48.0 HR 0.76 (95% CI 0.61-0.96) *P* = 0.01	1. Neutropenia (75.8%) 2. Leukopenia (31.3%) 3. Hot flush (26.3%)	37.0%^38^	2.2 months	
MONARCH-3^4^^,^^7^^,^^16^	Phase III RCT Abemaciclib + letrozole/letrozole	First line MBC	HR+/HER2− postmenopausal Locally recurrent metastatic with no prior ET ECOG PS 0/1	28.2 versus 14.8 HR 0.54 *P* = 0.00002	66.8 versus 53.7 HR 0.804 (95% CI 0.637-1.015) *P* = 0.03	1. Diarrhoea (82.3%) 2. Neutropenia (43.7%) 3. Fatigue (41.3%) and nausea (41.3%)	43.4%^41^	39.5 days	19.6%^41^
MONARCH-2^42^^,^^43^	Phase III RCT Abemaciclib + fulvestrant/Placebo + fulvestrant	Progressed on previous ET in adjuvant or metastatic setting	HR+/HER2− pre or postmenopausal ECOG PS 0/1	16.4 versus 9.3 HR 0.553 *P* = <0.001	46.7 versus 37.3 HR 0.757 (95% CI 0.606-0.945) *P* = 0.01	1. Diarrhoea (86.4%) 2. Neutropenia (46.0%) 3. Nausea (45.1%)	42.9%^42^	34 days^42^	15.9%^42^

CI, confidence interval; AI, aromatase inhibitor; CDK4/6i, cyclin-dependent kinase 4/6 inhibitor; ECOG PS, Eastern Cooperative Oncology Group performance status; ET, endocrine therapy; GnRHa, gonadotropin-releasing hormone agonist; HER2, human epidermal growth factor receptor 2; HR, hazard ratio; HR+, hormone receptor-positive; MBC, metastatic breast cancer; OS, overall survival; PFS, progression-free survival; RCT, randomised control trials.

The aim of this study was to describe, assess and compare the efficacy and tolerability of CDK4/6is across the Southwest region of the UK with trials and other RWD, through a retrospective cohort study including all patients treated with CDK4/6i for MBC at five centres between April 2017 and November 2022. A secondary aim was to investigate the effect of clinicopathological variables, treatment choice and DR on efficacy through univariate and multivariate analyses.

## Patients and methods

### Study design

**Figure 1:**
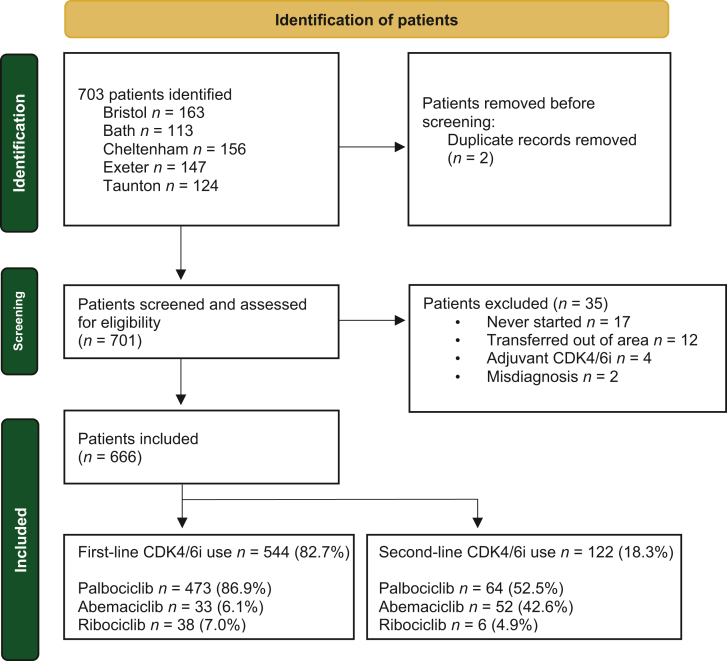
**Patient population and eligibility criteria.** A flowchart to show identification of patients, screening of patients and those included and excluded subjects. For those included, it illustrates the relative distribution of patients between first- and second-line CDKi use as well as by CDKi agent received. CDK4/6i, cyclin-dependent kinase 4/6 inhibitor.

This study used a retrospective cohort design to investigate the safety and efficacy of CDK4/6i as a first-line or subsequent-line treatment for MBC. All female patients with HR+/HER2− advanced or MBC who received at least one cycle of a CDK4/6i at five cancer centres in South West England (Bristol, Bath, Taunton, Cheltenham and Exeter) between April 2017 and November 2022 were included. With local institutional board approval for an audit process, meaning individual patient consent was not required, pseudonymised data were collected by manual review of electronic medical records (EMRs) (Figure 1). Local institutional policies were followed for data collection, storage and transfer. Data were collected using a universal, categorised Excel spreadsheet template to facilitate data input robustness and consistency. Data collected included baseline patient demographics, disease characteristics relating to advanced cancer and any previous early breast cancer diagnosis and treatment. First-line therapy was defined as the first systemic treatment initiated at the diagnosis of MBC. For those receiving CDK4/6i as a subsequent-line therapy, the number of prior systemic therapies and which agents they received for MBC were recorded. Outcomes to measure safety included toxicities, DRs and discontinuations, for which data were extracted by manual review of clinical notes. Toxicities were graded according to the Common Terminology Criteria for Adverse Events, either within the EMR or by investigator assessment of the free text clinical record. The number of cycles until DR was recorded, as well as any further DRs required. Outcomes to measure efficacy included PFS and OS. PFS was measured as the time from starting CDK 4/6i until disease progression, where possible taken as the date of radiological assessment demonstrating progression according to RECIST 1.1, or if unavailable the earliest date of clinical assessment of progression was used. Patients were followed up at regular intervals according to local institutional guidelines, typically with monthly review and three-monthly radiological assessment. OS was measured as the time from starting CDK4/6i until death due to any cause.

### Statistical analyses

To analyse efficacy outcomes, PFS and OS were examined in subgroups according to therapy line. Time-to-event outcomes were analysed using the Kaplan–Meier method. Within the first-line treatment subgroup, patients were grouped firstly by a CDK4/6 agent, and secondly by age quartile, and the log-rank test was used to compare outcomes between groups. Univariable and multivariable analyses were conducted in the first-line subgroup to evaluate the effect of treatment choice and clinicopathological variables on PFS and OS, using Cox regression for PFS and OS. Missing values were imputed using machine learning algorithms that implement multiple imputations by chained equations, using the R package ‘missRanger’ (v 2.4.0).^15^ The proportional hazards assumption was confirmed using the Schoenfeld residuals test. Baseline variables with a *P* value <0.05 in univariable analysis were included in the multivariable analysis. Sensitivity analyses were carried out by conducting univariable and multivariable models excluding patients with missing data, and the results were compared against those obtained from the imputed models. Analysis was carried out using R (version 4.3.2).

## Results

### Patient characteristics

Six hundred and sixty-six patients were included, with a median age of 66 years (range 23-92 years). Six hundred and seven (91.1%) had an Eastern Cooperative Oncology Group (ECOG) performance status (PS) score of 0-1 and 38 (5.7%) had a PS ≥2. Two hundred and forty-three (36.5%) patients were premenopausal, 344 (51.7%) were postmenopausal and 79 (11.9%) had no documented/unknown menopausal status. Most patients, 375 (56.3%), had visceral metastases, followed by bone-only metastases in 195 (29.3%) patients, non-visceral metastases in 41 (6.2%) patients and central nervous system disease in 11 (1.7%) patients. The site of metastatic disease was unknown or unrecorded in 44 (6.6% patients). Most patients, 487 (73.1%), had previously had an early breast cancer diagnosis, whilst 179 (26.9%) patients presented with *de novo* metastatic disease. For those who presented initially with early breast cancer, 278 (57.1%) had received prior neo-/adjuvant chemotherapy and 443 (91.0%) had neo-/adjuvant ET, of which 226 (46.4%) received tamoxifen and 180 (37.0%) had an aromatase inhibitor. This is summarised in Table 2.

**Table 2:** Baseline demographics and tumour characteristics for included patients at the time of CDK4/6i initiation

Treatment schedule	Palbociclib	Abemaciclib	Ribociclib	Total	*P* value^a^
Number of patients (% of total)	537 (80.6)	85 (12.8)	44 (6.6)	666 (100)	
Age					
Median (range) – years	65 (25-92)	70 (23-62)	62 (28-83)	66 (23-92)	
<65 years, *n* (%)	259 (48.2)	32 (37.6)	24 (54.5)	315 (47.3)	0.12
≥65 years, *n* (%)	278 (51.8)	53 (62.4)	20 (45.4)	351 (52.7)	
ECOG PS, *n* (%)					
0-1	490 (91.2)	79 (92.9)	38 (86.4)	607 (91.1)	0.24
2-3	28 (5.2)	5 (5.9)	5 (11.4)	38 (5.7)	
Unknown	19 (3.5)	1 (1.2)	1 (2.3)	21 (3.2)	
Menopausal status					
Premenopausal	190 (35.4)	33 (38.8)	20 (45.5)	243 (36.5)	0.19
Postmenopausal	289 (53.8)	35 (41.2)	20 (45.5)	344 (51.7)	
Unknown	58 (10.8)	17 (20.0)	4 (9.1)	79 (11.9)	
Disease site, *n* (%)					
Bone	165 (30.7)	16 (18.8)	14 (31.8)	195 (29.3)	0.24
Non-visceral	38 (7.1)	2 (2.4)	1 (2.3)	41 (6.2)	
Visceral	291 (54.2)	58 (68.2)	26 (59.1)	375 (56.3)	
CNS	8 (1.5)	3 (3.5)	0 (0)	11 (1.7)	
Other/unknown	35 (6.5)	6 (7.1)	3 (6.8)	44 (6.6)	
Metastatic at first diagnosis?					
Yes	155 (28.9)	10 (11.8)	14 (31.8)	179 (26.9)	0.003
No	382 (71.1)	75 (88.2)	30 (68.2)	487 (73.1)	
CDK4/6 setting					
First line	473 (88.1)	33 (38.8)	38 (86.4)	544 (81.7)	<0.001
Second line or beyond	64 (11.9)	52 (61.2)	6 (13.6)	122 (18.3)	
CDK4/6i ET backbone					
Letrozole	393 (73.2)	9 (10.6)	28 (63.6)	430 (64.6)	<0.001
Anastrozole	47 (8.8)	2 (2.4)	1 (2.3)	50 (7.5)	
Exemestane	16 (3.0)	0 (0)	0 (0)	16 (2.4)	
Fulvestrant	77 (14.3)	74 (87.1)	15 (34.1)	166 (24.9)	
Other	4 (0.7)	0 (0)	0 (0)	4 (0.6)	
Prior adjuvant or neoadjuvant Therapies, *n* (%)					
Prior chemotherapy	212 (39.5)	42 (49.4)	24 (54.5)	278 (41.7)	0.05
Neo-/adjuvant ET	350 (65.2)	69 (81.2)	24 (54.5)	443 (66.5)	0.03
Aromatase inhibitor	135 (25.1)	34 (40.0)	11 (25.0)	180 (27.0)	0.02
Tamoxifen	190 (35.4)	24 (28.2)	12 (27.3)	226 (34.0)	
Other	25 (4.7)	11 (12.9)	1 (2.3)	37 (5.6)	
Disease-free interval from adjuvant ET, *n* (% of those with EBC)	*n* = 382	*n* = 75	*n* = 30	*n* = 487	
≤12 months since adjuvant ET—first-line CDK4/6i	104 (27.2)	16 (21.3)	11 (36.7)	131 (26.9)	0.07
>12 months since adjuvant ET—first-line CDK4/6i	176 (46.1)	11 (14.7)	14 (46.7)	201 (41.3)	
Unknown	102 (26.7)	48 (64.0)	5 (16.7)	155 (31.8)	
Prior metastatic treatment (% of those treated with CDK4/6i second line and beyond)	*n* = 64	*n* = 52	*n* = 6	*n* = 122	
Hormone therapy	37 (57.8)	30 (57.7)	4 (66.7)	71 (58.2)	1
Chemotherapy	23 (35.9)	19 (36.5)	2 (33.3)	44 (36.1)	
Unknown/other	4 (6.3)	3 (5.8)	0 (0)	7 (5.7)	

CDK4/6i, cyclin-dependent kinase 4/6 inhibitor; CNS, central nervous system; EBC, early breast cancer; ECOG PS, Eastern Cooperative Oncology Group performance status; ET, endocrine therapy.

a Differences between groups were tested using the chi-square or Fisher’s test as appropriate.

### CDK4/6i treatment

Most patients received palbociclib (537 patients, 80.6%); 85 received abemaciclib (12.8%) and 44 received ribociclib (6.6%). The treatment groups appeared balanced in age, PS, menopausal status and disease site. There were imbalances regarding (neo)adjuvant treatments; patients receiving abemaciclib where significantly more likely to have received prior ET [69 patients (81.2%) abemaciclib; 350 patients (65.2%) palbociclib; 24 patients (54.5%) ribociclib; *P* = 0.003], and amongst these more had received an aromatase inhibitor [34 patients (40%) abemaciclib; 135 patients (25.1%) palbociclib; 11 patients (25.0%) ribociclib; *P* = 0.02]. There also appeared to be a trend towards fewer patients in the abemaciclib group who had relapsed >1 year after adjuvant ET [11 patients (14.7%) abemaciclib; 176 patients (46.1%) palbociclib; 14 patients (46.7%) ribociclib; *P* = 0.07], although there were substantial missing data for this variable. The proportion of patients with metastatic disease at initial breast cancer diagnosis differed between groups, with abemaciclib having the lowest proportion [10 patients (11.8%) abemaciclib; 155 patients (28.9%) palbociclib; 14 patients (31.8%) ribociclib; *P* = 0.003]. Abemaciclib was more frequently used in the second line (61.2% compared to 11.9% for palbociclib and 13.6% for ribociclib; *P* < 0.001) and was more frequently combined with fulvestrant [74 patients (87.1%) abemaciclib; 77 patients (14.3%) palbociclib; 15 patients (34.1%) ribociclib; *P* < 0.001] (Table 2).

### Side-effects

Overall, any-grade toxicity was reported in 527 (79.2%) patients. This differed by CDK4/6i received [414 patients (77.2%) receiving palbociclib, 72 patients (84.7%) receiving abemaciclib and 41 patients (93.1%) receiving ribociclib]. Grade 3 or worse toxicity was documented overall in 248 patients (37.2%): 203 patients (37.9%) receiving palbociclib, 22 patients (25.9%) receiving abemaciclib and 23 patients (52.3%) receiving ribociclib (Table 3).

**Table 3:** Side-effects

Toxicity	Palbociclib (*n* = 537)		Abemaciclib (*n* = 85)		Ribociclib (*n* = 44)	
	Any grade (%)	≥G3 (%)	Any grade (%)	≥G3 (%)	Any grade (%)	≥G3 (%)
Neutropenia	38.2	30.3	15.3	7.1	36.4	15.9
Fatigue	37.1	1.9	31.8	2.4	34.1	4.5
Diarrhoea	9.1	0.7	61.2	10.6	15.9	2.3
Mucositis	12.1	0.6	4.8	0	2.3	0
Hepatotoxicity	2.8	1.3	1.2	1.2	27.3	20.5
Nausea	9.9	1.3	17.6	5.9	25.0	2.3
Rash	3.2	0	2.4	0	9.1	2.3
Anaemia	3	1.1	2.4	0	2.3	0
Thrombocytopenia	3.2	1.9	2.4	1.2	2.3	2.3
Anorexia	1.3	0	3.5	0	2.3	0
Vomiting	0.2	0	8.2	2.4	0	0

Most common side-effects of both any grade and ≥ grade 3 by the different CDK4/6is are reported as percentages.

CDK4/6i, cyclin-dependent kinase 4/6 inhibitor; G3, grade 3.

In the palbociclib and ribociclib group the most common any-grade side-effects were neutropenia [205 patients (38.2%) and 16 patients (36.4%), respectively] followed by fatigue in [199 patients (37.1%) and 15 patients (34.1%) respectively]. The most common any-grade toxicity for patients receiving abemaciclib was diarrhoea in 52 patients (61.2%), followed by fatigue in 27 patients (31.8%). With regard to grade 3 or 4 toxicity, for palbociclib neutropenia was the most common [163 patients (30.3%)]. Diarrhoea, which was seen in five patients (10.6%), and hepatotoxicity affecting nine patients (20.5%) were the most common grade 3 or 4 toxicities for abemaciclib and ribociclib, respectively.

### Dose reductions (DRs) and dose delays

DRs were similar across the three CDK4/6is. DRs were noted in 289 patients (53.8%) receiving palbociclib, 50 patients (58.8%) receiving abemaciclib and 26 patients (59.1%) receiving ribociclib (Table 4). The median time to first DR was two cycles for ribociclib and three cycles for those taking palbociclib and abemaciclib. Permanent discontinuation of CDK4/6i occurred in 37 patients (6.9%), 17 patients (20.0%) and 11 patients (25.0%) receiving palbociclib, abemaciclib and ribociclib, respectively.

**Table 4:** Dose & toxicity data

	Palbociclib	Abemaciclib	Ribociclib
Number of patients (%)	537 (80.6)	85 (12.8)	44 (6.6)
Number of patients who had dose reduction (%)	289 (53.8)	50 (58.8)	26 (59.1)
Median number of cycles before first dose reduction (range)	3 (1-63)	3 (1-11)	2 (1-37)
Permanent discontinuation due to toxicity, *n* (%)	37 (6.9)	17 (20.0)	11 (25.0)

Table summarises the number and percentage of patients who had a dose reduction, median time to first dose reduction and continuation rates for all patients in the cohort broken down by CDK4/6i agent.

CDK4/6i, cyclin-dependent kinase 4/6 inhibitor.

### CDK4/6i PFS—first-line setting

**Figure 2:**
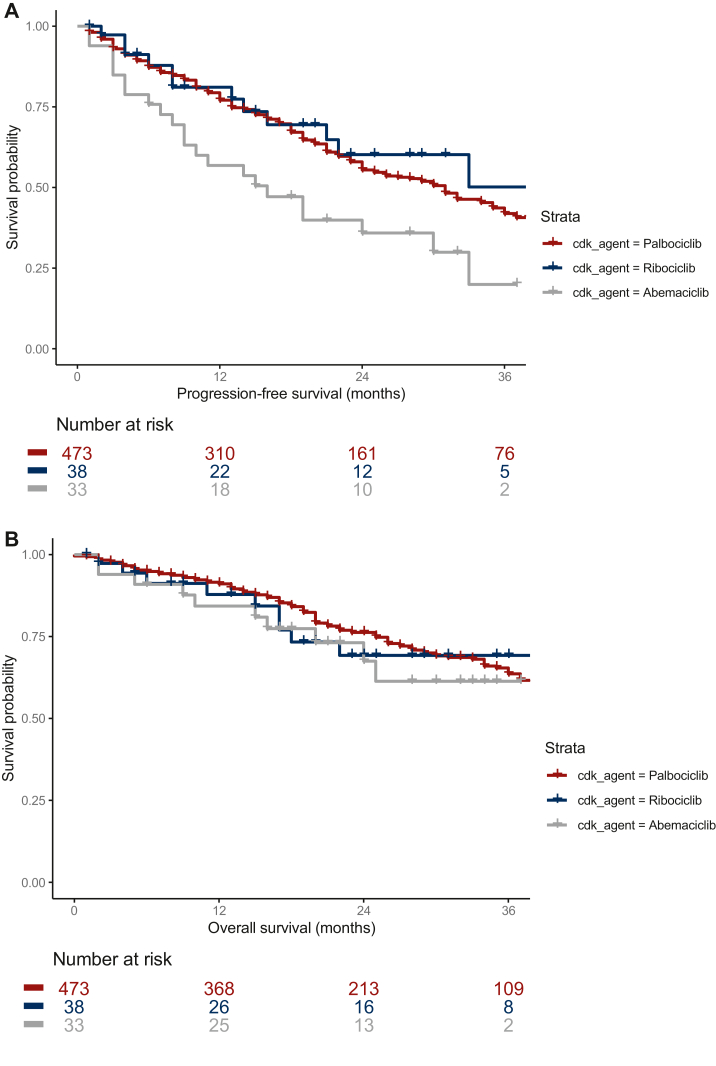
**PFS and OS in Kaplan-Meier analysis for 1st line CDK4/6i use (36 month follow-up).** (A) Kaplan–Meier analysis of PFS until 36 months follow-up for all patients in the cohort receiving CDK4/6 inhibitors as a first-line therapy for ER+/HER2− MBC. (B) Kaplan–Meier analysis of OS until 36 months follow-up for all patients in the cohort receiving CDK4/6 inhibitors as a first-line therapy for ER+/HER2− MBC. CDK4/6, cyclin-dependent kinase 4/6; ER, estrogen receptor; HER2, human epidermal growth factor receptor 2; MBC, metastatic breast cancer; OS, overall survival; PFS, progression-free survival.

The median follow-up time was 28 months (range 0-76 months) and was consistent between treatment groups (median 29 months for palbociclib, 27 months for ribociclib and 28 months for abemaciclib). For patients receiving CDK4/6i as first-line treatment, PFS was shorter with abemaciclib [median 16 months, 95% confidence interval (CI) 9 months-not reached (NR)] than palbociclib (median 31 months, 95% CI 25-35 months) or ribociclib (median 44 months, 95% CI 21 months-NR), log-rank *P* = 0.015 for the three-group comparison (Figure 2A). Follow-up until 36 months is shown in Figure 2, after which follow-up data for patients receiving abemaciclib and ribociclib were limited. Multivariable analysis (Supplementary Table S2, available at https://doi.org/10.1016/j.esmorw.2024.100064) showed that abemaciclib was not significantly associated with PFS when controlling for potential confounders [hazard ratio (HR) 1.46, 95% CI 0.87-2.46, *P* = 0.15].

Age had a significant effect on PFS in the multivariable model, with older patients having a lower risk of progression (HR 0.98, 95% CI 0.97-0.99, *P* = 0.002). A further analysis examining PFS by age quartiles showed that the shorter PFS observed with reduced age was driven by the youngest patients (Supplementary Figure S1A, available at https://doi.org/10.1016/j.esmorw.2024.100064). In univariate analysis age had no significant effect on OS (HR 1.01, 95% CI 0.99-1.02, *P* = 0.5) (Supplementary Figure S1B, available at https://doi.org/10.1016/j.esmorw.2024.100064).

Patients with visceral metastases had a shorter PFS than those with bone-only metastatic disease in multivariate analysis (HR 1.58, 95% CI 1.19-2.09, *P* = 0.001). Patients who had a DR of CDK4/6i had a significantly longer PFS on multivariate analysis (HR 0.74, 95% CI 0.58-0.96, *P* = 0.02) compared to those who had no DR. Patients receiving fulvestrant as anti-estrogen backbone had shorter PFS than those receiving letrozole on multivariate analysis (HR 1.60, 95% CI 1.07-2.39, *P* = 0.02); however, this correlation is potentially subject to confounding. The fulvestrant group was enriched for patients who had prior treatment with an aromatase inhibitor and/or relapsed at or within 12 months of adjuvant ET. These variables were not included in the multivariable model because they were only measured in the subset of patients who had previously presented with early breast cancer. Complete case analysis (Supplementary Table S4, available at https://doi.org/10.1016/j.esmorw.2024.100064) showed comparable results to the imputed model.

### CDK4/6i OS—first-line setting

OS was comparable between the three CDK4/6i agents; median OS was 47 months (41 months-NR) for palbociclib and was not reached for either abemaciclib or ribociclib (log-rank *P* = 0.61) (Figure 2B). Hazard ratio (HR) for OS with abemaciclib compared with palbociclib was 1.33 (95% CI 0.7-2.53, *P* = 0.39). In multivariable analysis, ECOG PS, sites of metastatic disease, anti-estrogen backbone and DRs had significant effects on OS (Supplementary Table S3, available at https://doi.org/10.1016/j.esmorw.2024.100064). Shorter OS was observed with a PS of 2 or more (HR 2.01, 95% CI 1.13-3.58, *P* = 0.02), and with visceral compared with bone-only metastatic disease (HR 1.61, 95% CI 1.13-2.30, *P* = 0.009). OS appeared shorter for those receiving fulvestrant compared with letrozole (HR 2.04, 95% CI 1.35-3.10, *P* < 0.001). However, this could be confounded by an excess of patients who had prior treatment with an aromatase inhibitor and/or relapsed at or within 12 months of adjuvant ET. DR appeared to be strongly associated with improved OS (HR 0.52, 95% CI 0.38-0.72, *P* < 0.001). Complete case analysis (Supplementary Table S5, available at https://doi.org/10.1016/j.esmorw.2024.100064) showed comparable results to the imputed model.

### CDK4/6i PFS and OS—second-line or beyond setting

For patients treated with a CDK4/6i in the second-line or beyond setting, the median PFS was 12 months (95% CI 10-29 months) for palbociclib, 15 months (95% CI 11-30 months) for abemaciclib and 15.5 months (95% CI 10 months-NR) for ribociclib (Supplementary Figure S2A, available at https://doi.org/10.1016/j.esmorw.2024.100064). Median OS for patients treated in the second line or beyond was 25 months for palbociclib (95% CI 14-50 months), 24 months for abemaciclib (95% CI 24 months-NR) and was not reached for ribociclib (Supplementary Figure S2B, available at https://doi.org/10.1016/j.esmorw.2024.100064).

## Discussion

This multicentre real-world study including 666 patients demonstrates that CDK4/6is are safe and effective outside of a trial setting, with findings consistent with randomised control trials (RCTs) and other RWD (Supplementary Table S1, available at https://doi.org/10.1016/j.esmorw.2024.100064).^9^, ^10^, ^11^, ^12^, ^13^^,^^16^, ^17^, ^18^ Firstly, an association between increasing age and longer PFS, underscoring that CDK4/6is are effective in older patients, was demonstrated. Secondly, DRs were strongly associated with longer PFS and OS, underlining the need to prospectively evaluate lower starting doses of CDK4/6i.

Most patients within this cohort received palbociclib (80%), reflecting clinical practice at the time of data collection. Recent trial data potentially support the use of ribociclib and abemaciclib^19^, ^20^, ^21^ over palbociclib, and this could lead to a change in prescribing patterns. The widespread use of CDK4/6i as adjuvant therapy for early breast cancer^22^,^23^ will shape treatment patterns further, as patients begin to relapse after the use of early-stage adjuvant CDK4/6i.

The observed relationship between older age and longer PFS in this cohort is consistent with other large RWD studies.^14^^,^^24^ In this cohort, the effect was driven by a shorter PFS in the youngest age quartile. This could be explained by an enrichment of germline or somatic genetic variants associated with inferior outcomes; however, these were not measured in our study. For example, younger patients have a higher prevalence of germline pathogenic variants such as g*BRCA*1/2, *ATM* and *CHEK2,* which can negatively impact PFS and OS with CDK4/6i.^25^ Furthermore, higher rates of somatic MAPK/PI3K variants are observed in younger patients, which also confer a worse prognosis in estrogen receptor-positive breast cancer.^26^ Further studies investigating the efficacy of CDK4/6i in molecular subtypes of MBC would be beneficial.^20^^,^^24^

The majority of patients in our cohort required a DR for toxicity, irrespective of which CDK4/6i they received. DR was strongly associated with improvements in both PFS and OS on multivariate analysis. The rates of DR and apparent positive effect on outcome are in line with other RWDs.^10^^,^^11^ DRs may lead to improved tolerability, fewer drug delays and interruptions, permitting a more constant plasma drug level.^11^ However, this relationship could be overestimated in non-randomised data, which are subject to bias from patients who had early disease progression leading to a systemic treatment agent change without a DR.^27^ Prospective trials evaluating lower starting doses in the metastatic setting are warranted to establish whether CDK4/6i starting doses can be safely lowered without compromising efficacy. Lower doses are effective in the adjuvant setting,^23^ and appear effective in subgroup analyses of patients with DRs within both adjuvant and metastatic trials.^23^^,^^28^

The toxicity profile in our cohort was in line with RCTs and RWDs. As expected, the most common toxicity was neutropenia in patients receiving palbociclib or ribociclib, or diarrhoea in patients receiving abemaciclib. Numerically, our data appear to show lower rates of recorded toxicity compared to RCT data, although this was not formally tested. There could be under-reporting or less-stringent documentation in a real-world setting. In RWD, it is acknowledged that lower rates of toxicity reflect the challenge of retrospective medical note reviews compared to the rigorous prospective data collection that takes place within RCTs.^29^ Permanent discontinuation rates due to toxicity were in line with other data^9^^,^^10^ but appeared less frequent with palbociclib than abemaciclib or ribociclib. This may reflect clinicians’ confidence and experience in managing neutropenia, compared to the diarrhoea and hepatotoxicity observed with abemaciclib and ribociclib, respectively.

The efficacy outcomes in our study were consistent with those reported in RCTs and other RWDs. The rate of *de novo* metastatic disease was 27% in our study, which is comparable to that observed in other similar RWD data.^12^^,^^17^ Visceral metastases and ECOG PS 2-3 were associated with shorter PFS and OS on multivariate analysis. A shorter PFS was noted in patients treated with abemaciclib, which might be explained by a higher incidence of endocrine resistance among these patients as most had received prior ET (82%) and were treated with abemaciclib in combination with fulvestrant (87%), suggesting endocrine resistance.^30^

This study has limitations inherent in the retrospective design. Follow-up time was short meaning that median OS was not reached, and data were collected from only five centres from one geographical region. Fewer patients were treated with ribociclib and abemaciclib, making this a relatively small dataset for comparative efficacy. There were unbalanced baseline characteristics between the groups (Table 2), including an excess of ET-resistant patients receiving abemaciclib/fulvestrant, which could have confounded the multivariable analysis. Approaches to correct for indication bias including propensity score matching and interaction analysis were considered but given the small numbers of patients receiving abemaciclib and ribociclib, the value would be limited. Multiple imputation was carried out to mitigate potential biases from missing data in the baseline variables. Complete case analysis showed consistent results, suggesting that missing data were not a significant source of bias.

### Conclusion

These data demonstrate that CDK4/6is are an effective and safe treatment for HR+/HER2− MBC in a heterogenous, real-world population across several cancer centres. They add to growing evidence supporting prospective evaluation of lower dose CDK4/6i in MBC. The integration of adjuvant CDK4/6i as the standard care in HR+/HER2- early breast cancer will significantly impact frontline treatment of MBC in the future, as many patients will relapse with prior exposure to CDK4/6i. RWD will be increasingly important to evaluate the benefits of CDK4/6i in this new context.
