# Supplementary material for: UK multicentre real-world data of the use of cyclin-dependent kinase 4/6 inhibitors in metastatic breast cancer
Source: ESMO Real World Data Digit Oncol. 2024 Aug 20;5:100064. doi: 10.1016/j.esmorw.2024.100064 (PMC12836663; doi:10.1016/j.esmorw.2024.100064)
Supplement: Supplementary Table 1 [file mmc1.pdf]

Supplementary Table 1. A summary of RWD publications examining the use of CDK4/6i in >200 patients with HR+/HER2- advanced breast cancer

1L treatment, first line treatment; PFS, progression-free survival; CI, confidence interval; OS, overall survival; AI, aromatase inhibitor; CDK4/6i, CDK4/6 inhibitor; NR, not reached; NE, not evaluable.

Median PFS, median OS, dose reduction and discontinuation rates are reported by individual CDK4/6i agents where comparative analyses were performed. 95% CI for PFS and OS reported in brackets, where available.

**These studies did not perform comparative analysis by CDK4/6i agent; median PFS, median OS, dose reduction and discontinuation rates are reported for entire cohort, or by endocrine therapy subtype where specified in brackets.*

Study	Country	Study design	Patient population	Treatment	Number of patients	1L Median PFS, months (95% CI)	1L Median OS (months)	Dose reduction	Discontinued due to toxicity
DeMichele<br>Breast Cancer Res<br>2021[16]	US	Retrospective observational analysis	1L treatment, Palbociclib +/- AI	Palbociclib + Letrozole	772	19.7<br>(17.3–21.9)	NR	-	-
Low<br>The Adv Med Oncol<br>2022[10]	Singapore	Multicentre cohort study	Any line<br>CDK4/6i + ET	Palbociclib + ET<br>Ribociclib + ET	435<br>21	28.2	-	48%	-
Muller<br>Breast care<br>2023[11]	Germany	Multicentre retrospective study	Any line<br>CDK4/6i + ET	Palbociclib + ET<br>Ribociclib + ET<br>Abemaciclib + ET	319<br>114<br>15	23	-	29%	13%
Fountzilas<br>ESMO Open<br>2020[17]	Greece and Cyprus	Multicentre retrospective analysis	Any line<br>CDK4/6i + ET	Palbociclib + ET<br>Ribociclib + ET	301<br>64	18.7 (13.5-NE)	NR	14%	6%
Buller<br>J Oncol Pharm Pract.<br>2023[9]	UK	Single centre retrospective study	Any line<br>CDK4/6i + ET	Palbociclib + ET<br>Ribociclib + ET<br>Abemaciclib + ET	136<br>37<br>56	27.9 (23-32.5)<br>29 (21.5-37.0)<br>20.6 (15.0-26.0)	38.0 (33.5-42.5)<br>33.9 (26.7-41.1)<br>27.3 (22.5-32.1)	54%<br>57%<br>41%	2%<br>5%<br>-
Tang<br>Cancers<br>2023[13]	UK	Single centre retrospective study	1L treatment,<br>CDK4/6i + AI,<br>post-menopausal	Palbociclib + AI<br>Ribociclib + AI<br>Abemaciclib + AI	162<br>46<br>19	27.5<br>25.7<br>NR	49.5<br>50.2<br>NR	-	-
Knudsen<br>Oncologist<br>2022[18]	US	Single centre retrospective study	Any line<br>CDK4/6i + ET	Palbociclib + ET<br>Ribociclib + ET<br>Abemaciclib + ET	208<br>7<br>7	27.6 (CDK4/6i + AI)<br>17.2 (CDK4/6i + Fulvestrant)	-	26%	-
Cejuela<br>Int J Mol Sci.<br>2023[12]	Spain	Single centre retrospective study	1L treatment<br>CDK4/6i + ET	Palbociclib + ET<br>Ribociclib + ET<br>Abemaciclib + ET	96<br>54<br>56	30.1<br>31.1<br>39.5	NR<br>NR<br>NR	52%<br>52%<br>54%	-<br>-<br>-
