# Supplementary material for: UK multicentre real-world data of the use of cyclin-dependent kinase 4/6 inhibitors in metastatic breast cancer
Source: ESMO Real World Data Digit Oncol. 2024 Aug 20;5:100064. doi: 10.1016/j.esmorw.2024.100064 (PMC12836663; doi:10.1016/j.esmorw.2024.100064)
Supplement: Supplementary Table 2 [file mmc2.pdf]

Supplementary Table 2: Univariable and Multivariable Cox-Proportional hazard model for PFS of patients receiving CDK4/6i in 1st line setting.

CDK4/6i, cyclin-dependent kinase 4/6 inhibitor; PFS, progression-free survival; HR, hazard ratio; 95%CI LL, 95% confidence interval lower limit; 95%CI UL 95% confidence interval upper limit; ECOG PS, Eastern Cooperative Oncology Group Performance Status; n, number

Variables "Prior adjuvant or neoadjuvant therapies" and "Disease free interval from adjuvant" were not included in the multivariable analysis as they refer to a subset of patients from the entire cohort, those who had previously been treated for early breast cancer. Missing values were imputed using MICE.

	**Subgroup**	**n**	**Univariable**					**Multivariable**				
			HR	95%CI LL	95% CI UL	P-value (relationship)	P-value (model)	HR	95%CI LL	95% CI UL	P-value (relationship)	P-value (overall for variable)
**CDK4/6i**	**Palbociclib**	473	1				**0.02**	1				**0.03**
	**Ribociclib**	38	0.91	0.53	1.57	0.74		0.86	0.50	1.49	0.60	
	**Abemaciclib**	33	1.88	1.21	2.92	0.005		1.46	0.87	2.46	0.15	
**Age**	**n/a**	n/a	0.99	0.98	0.996	**0.006**	**0.006**	0.98	0.97	0.99	**0.002**	**0.002**
**ECOG**	**0-1**	516	1				0.1					
	**2+**	28	1.51	0.89	2.54	0.13						
**Menopausal status**	**Post-menopausal**	318	1				0.4					
	**Pre-menopausal**	226	1.11	0.86	1.43	0.41						
**Metastatic at diagnosis**	**No**	389	1				0.9					
	**Yes**	155	0.99	0.76	1.3	0.9						
**Previous anti-oestrogen therapy**	**No**	179	1				0.4					
	**Yes**	365	1.12	0.86	1.46	0.41						
**Metastatic sites**	**Bone**	187	1				**0.03**	1				**0.01**
	**Non-visceral**	36	1.09	0.61	1.93	0.77		1.04	0.58	1.87	0.89	
	**Visceral**	318	1.50	1.13	1.98	0.004		1.58	1.19	2.09	0.001	
	**CNS**	3	0.63	0.09	4.56	0.65		0.60	0.08	4.36	0.62	
**Anti-oestrogen backbone**	**Letrozole**	396	1				**0.02**	1				0.14
	**Anastrazole**	43	1.22	0.76	1.96	0.42		1.35	0.83	2.19	0.23	
	**Exemestane**	14	0.86	0.35	2.1	0.74		0.88	0.36	2.16	0.78	
	**Fulvestrant**	88	1.68	1.19	2.36	0.003		1.60	1.07	2.39	0.02	
	**Other**	3	3.27	0.81	13.28	0.10		1.93	0.47	8.01	0.36	
**CDK4/6 inhibitor dose reduction**	**No**	235	1				**0.005**	1				0.02
	**Yes**	309	0.70	0.55	0.90	0.005		0.74	0.58	0.96	0.02	
