# Supplementary material for: UK multicentre real-world data of the use of cyclin-dependent kinase 4/6 inhibitors in metastatic breast cancer
Source: ESMO Real World Data Digit Oncol. 2024 Aug 20;5:100064. doi: 10.1016/j.esmorw.2024.100064 (PMC12836663; doi:10.1016/j.esmorw.2024.100064)
Supplement: Supplementary Table 3 [file mmc3.pdf]

Supplementary Table 3: Univariable and Multivariable Cox-Proportional hazard model for OS of patients receiving CDK4/6i in 1st line setting

CDK4/6i, cyclin-dependent kinase 4/6 inhibitor; OS, overall survival; HR, hazard ratio; 95%CI LL, 95% confidence interval lower limit; 95%CI UL 95% confidence interval upper limit; ECOG PS, Eastern Cooperative Oncology Group Performance Status; n, number

Variables "Prior adjuvant or neoadjuvant therapies" and "Disease free interval from adjuvant" were not included in the multivariable analysis as they refer to a subset of patients from the entire cohort, those who had previously been treated for early breast cancer. Missing values were imputed using MICE.

	**Subgroup**	**n**	**Univariable**					**Multivariable**				
			HR	95%CI LL	95% CI UL	P-value (relationship)	p-value (model)	HR	95%CI LL	95% CI UL	P-value (relationship)	p-value (overall for variable)
**CDK4/6i**	**Palbociclib**	473	1				0.6					
	**Ribociclib**	38	0.85	0.44	1.68	0.65						
	**Abemaciclib**	33	1.33	0.7	2.53	0.39						
**Age**	**n/a**	n/a	1.01	0.99	1.02	0.5	0.5					
**ECOG**	**0-1**	516	1				**0.008**	1				**0.02**
	**2+**	28	2.16	1.22	3.81	0.006		2.01	1.13	3.58	0.02	
**Menopausal status**	**Post-menopausal**	318	1				0.3					
	**Pre-menopausal**	226	0.85	0.62	1.16	0.31						
**Metastatic at diagnosis**	**No**	389	1				0.3					
	**Yes**	155	0.83	0.58	1.18	0.3						
**Previous anti-oestrogen therapy**	**No**	179	1				**0.3**					
	**Yes**	365	1.20	0.86	1.70	0.29						
**Metastatic sites**	**Bone**	187	1				**0.05**	1				**0.04**
	**Non-visceral**	36	1.09	0.51	2.32	0.83		1.01	0.47	2.20	0.97	
	**Visceral**	318	1.60	1.12	2.28	0.009		1.61	1.13	2.30	0.009	
	**CNS**	3	1.06	0.15	7.72	0.96		1.02	0.14	7.47	0.99	
**Anti-oestrogen backbone**	**Letrozole**	396	1				**0.004**	1				**0.008**
	**Anastrazole**	43	1.31	0.72	2.38	0.38		1.40	0.76	2.57	0.28	
	**Exemestane**	14	1.71	0.75	3.89	0.20		1.33	0.58	3.07	0.50	
	**Fulvestrant**	88	2.12	1.4	3.2	<0.001		2.04	1.35	3.10	<0.001	
	**Other**	3	3.06	0.75	12.43	0.12		1.84	0.41	8.29	0.43	
**CDK4/6 inhibitor dose reduction**	**No**	235	1				**<0.001**	1				**<0.001**
	**Yes**	309	0.49	0.36	0.66	<0.001		0.52	0.38	0.72	<0.001	
