# Supplementary material for: UK multicentre real-world data of the use of cyclin-dependent kinase 4/6 inhibitors in metastatic breast cancer
Source: ESMO Real World Data Digit Oncol. 2024 Aug 20;5:100064. doi: 10.1016/j.esmorw.2024.100064 (PMC12836663; doi:10.1016/j.esmorw.2024.100064)
Supplement: Supplementary Table 4 [file mmc4.pdf]

Supplementary Table 4: Univariable and Multivariable Cox-Proportional hazard models for PFS of patients receiving CDK4/6i in 1st line setting, by complete case analysis, for comparison with imputed data as sensitivity analysis

CDK4/6i, cyclin-dependent kinase 4/6 inhibitor; PFS, progression-free survival; HR, hazard ratio; 95%CI LL, 95% confidence interval lower limit; 95%CI UL 95% confidence interval upper limit; ECOG PS, Eastern Cooperative Oncology Group Performance Status; n, number

	**Subgroup**	**n**	**Univariable**						**n**	**Multivariable**				
			HR	95%CI LL	95% CI UL	P-value (relationship)	P-value (model)			HR	95%CI LL	95% CI UL	P-value (relationship)	P-value (overall for variable)
**CDK4/6i**	**Palbociclib**	473	1				**0.02**		401	1				**0.006**
	**Ribociclib**	38	0.91	0.53	1.57	0.74			32	0.84	0.48	1.48	0.54	
	**Abemaciclib**	33	1.88	1.21	2.92	0.005			25	1.63	0.97	2.74	0.06	
**Age**	**n/a**	n/a	0.99	0.98	0.996	**0.006**	**0.006**			0.98	0.97	0.99	**<0.001**	**<0.001**
**ECOG**	**0-1**	508	1				0.1		432					
	**2+**	28	1.54	0.91	2.59	0.11			26					
**Menopausal status**	**Post-menopausal**	299	1				0.4		181					
	**Pre-menopausal**	191	1.12	0.86	1.46	0.39			277					
**Metastatic at diagnosis**	**No**	389	1				0.9		314					
	**Yes**	155	0.99	0.76	1.3	0.9			144					
**Previous anti-oestrogen therapy**	**No**	174	1				0.4		160					
	**Yes**	350	1.12	0.85	1.46	0.43			298					
**Metastatic sites**	**Bone**	172	1				**0.03**		145	1				**0.003**
	**Non-visceral**	36	1.14	0.64	2.04	0.65			28	1.06	0.58	1.94	0.85	
	**Visceral**	298	1.63	1.23	2.18	0.001			254	1.73	1.28	2.33	<0.001	
	**CNS**	3	0.67	0.09	4.81	0.65			1	0.65	0.09	4.70	0.67	
**Anti-oestrogen backbone**	**Letrozole**	396	1				**0.02**		335	1				0.11
	**Anastrazole**	43	1.22	0.76	1.96	0.42			34	1.40	0.85	2.28	0.18	
	**Exemestane**	14	0.86	0.35	2.1	0.74			13	1.24	0.50	3.06	0.64	
	**Fulvestrant**	88	1.68	1.19	2.36	0.003			74	1.69	1.12	2.53	0.01	
	**Other**	3	3.27	0.81	13.28	0.10			2	1.18	0.16	8.60	0.87	
**CDK4/6 inhibitor dose reduction**	**No**	228	1				**0.01**		191	1				0.04
	**Yes**	302	0.72	0.56	0.92	0.01			267	0.76	0.58	0.98	0.04	
