# Supplementary material for: UK multicentre real-world data of the use of cyclin-dependent kinase 4/6 inhibitors in metastatic breast cancer
Source: ESMO Real World Data Digit Oncol. 2024 Aug 20;5:100064. doi: 10.1016/j.esmorw.2024.100064 (PMC12836663; doi:10.1016/j.esmorw.2024.100064)
Supplement: Supplementary Table 5 [file mmc5.pdf]

Supplementary Table 5: Univariable and Multivariable Cox-Proportional hazard models for OS of patients receiving CDK4/6i in 1st line setting, by complete case analysis, for comparison with imputed data as sensitivity analysis

CDK4/6i, cyclin-dependent kinase 4/6 inhibitor; PFS, progression-free survival; HR, hazard ratio; 95%CI LL, 95% confidence interval lower limit; 95%CI UL 95% confidence interval upper limit; ECOG PS, Eastern Cooperative Oncology Group Performance Status; n, number

	**Subgroup**	**n**	**Univariable**						**n**	**Multivariable**				
			HR	95%CI LL	95% CI UL	P-value (relationship)	P-value (model)			HR	95%CI LL	95% CI UL	P-value (relationship)	P-value (overall for variable)
**CDK4/6i**	**Palbociclib**	473	1				0.6		401					
	**Ribociclib**	38	0.85	0.44	1.68	0.65			32					
	**Abemaciclib**	33	1.33	0.70	2.53	0.39			25					
**Age**	**n/a**	n/a	1.01	0.99	1.02	0.5	0.5							
**ECOG**	**0-1**	508	1				**0.006**		432	1				**0.01**
	**2+**	28	2.24	1.26	3.96	0.006			26	1.99	1.11	3.56	0.02	
**Menopausal status**	**Post-menopausal**	299	1				0.2		181					
	**Pre-menopausal**	191	0.81	0.57	1.13	0.22			277					
**Metastatic at diagnosis**	**No**	389	1				0.3		314					
	**Yes**	155	0.83	0.58	1.18	0.3			144					
**Previous anti-oestrogen therapy**	**No**	174	1				0.3		160					
	**Yes**	350	1.20	0.84	1.70	0.31			298					
**Metastatic sites**	**Bone**	172	1				**0.03**		145	1				0.06
	**Non-visceral**	36	1.15	0.53	2.46	0.72			28	0.99	0.44	2.26	0.99	
	**Visceral**	298	1.75	1.20	2.54	0.003			254	1.62	1.10	2.37	0.01	
	**CNS**	3	1.09	0.15	7.96	0.93			1	1.02	0.14	7.49	0.99	
**Anti-oestrogen backbone**	**Letrozole**	396	1				**0.004**		335	1				**0.005**
	**Anastrazole**	43	1.31	0.72	2.38	0.38			34	1.45	0.74	2.84	0.27	
	**Exemestane**	14	1.71	0.75	3.89	0.20			13	2.14	0.92	4.98	0.08	
	**Fulvestrant**	88	2.12	1.4	3.2	<0.001			74	2.12	1.37	3.27	<0.001	
	**Other**	3	3.06	0.75	12.43	0.12			2	1.78	0.24	12.91	0.57	
**CDK4/6 inhibitor dose reduction**	**No**	228	1				**0.01**		191	1				**<0.001**
	**Yes**	302	0.50	0.36	0.68	<0.001			267	0.53	0.38	0.74	<0.001	
