# Supplementary material for: UK multicentre real-world data of the use of cyclin-dependent kinase 4/6 inhibitors in metastatic breast cancer
Source: ESMO Real World Data Digit Oncol. 2024 Aug 20;5:100064. doi: 10.1016/j.esmorw.2024.100064 (PMC12836663; doi:10.1016/j.esmorw.2024.100064)

Supplementary Figure 1: (A) Kaplan-Meier subgroup analysis of PFS by age quartiles for all patients in the cohort receiving CDK4/6 inhibitors as a first line therapy for ER+/HER2- MBC. (B) Kaplan-Meier subgroup analysis of OS by age quartiles for all patients in the cohort receiving CDK4/6 inhibitors as a first line therapy for ER+/HER2- MBC. p-value calculated by log-rank test

3A

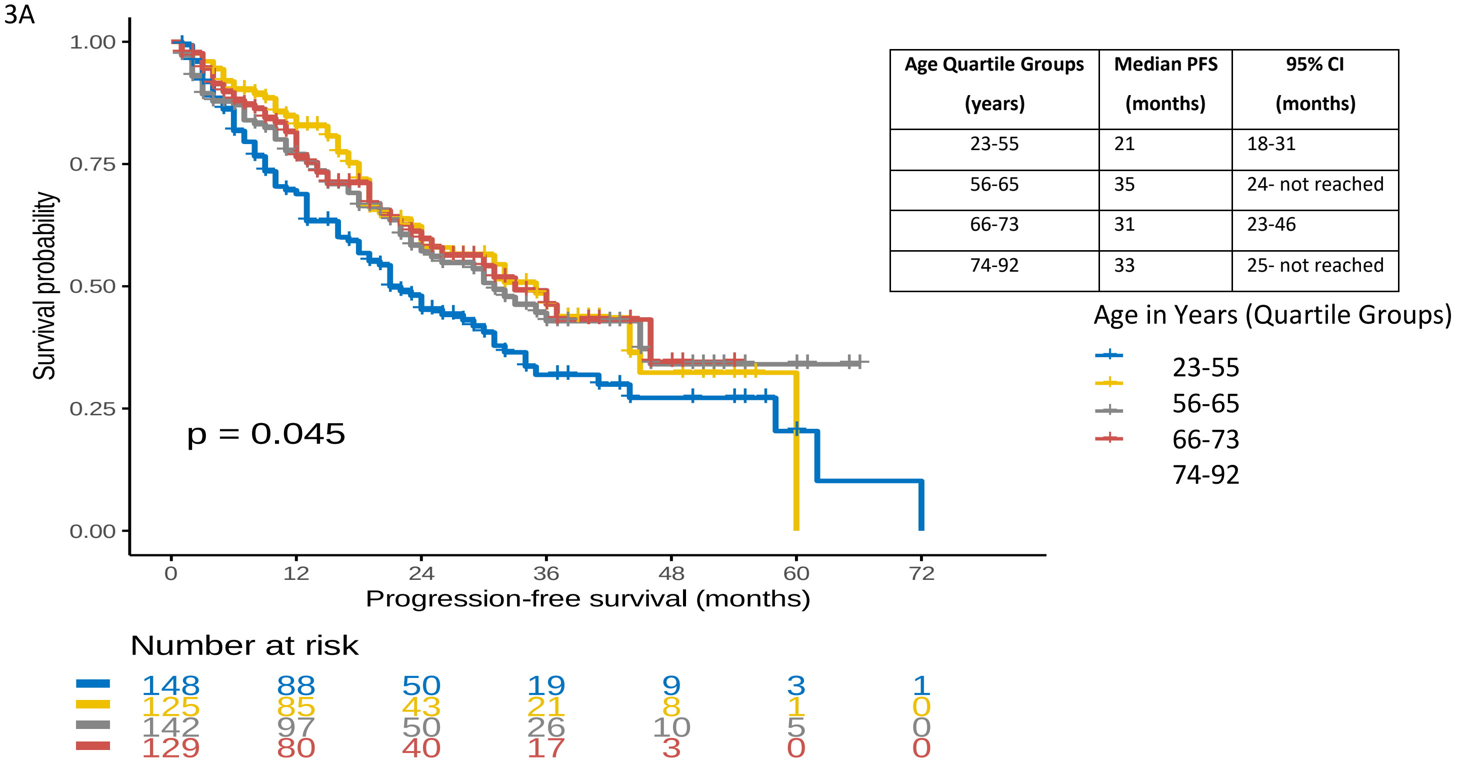

Age Quartile Groups (years)	Median PFS (months)	95% CI (months)
23-55	21	18-31
56-65	35	24- not reached
66-73	31	23-46
74-92	33	25- not reached

3B

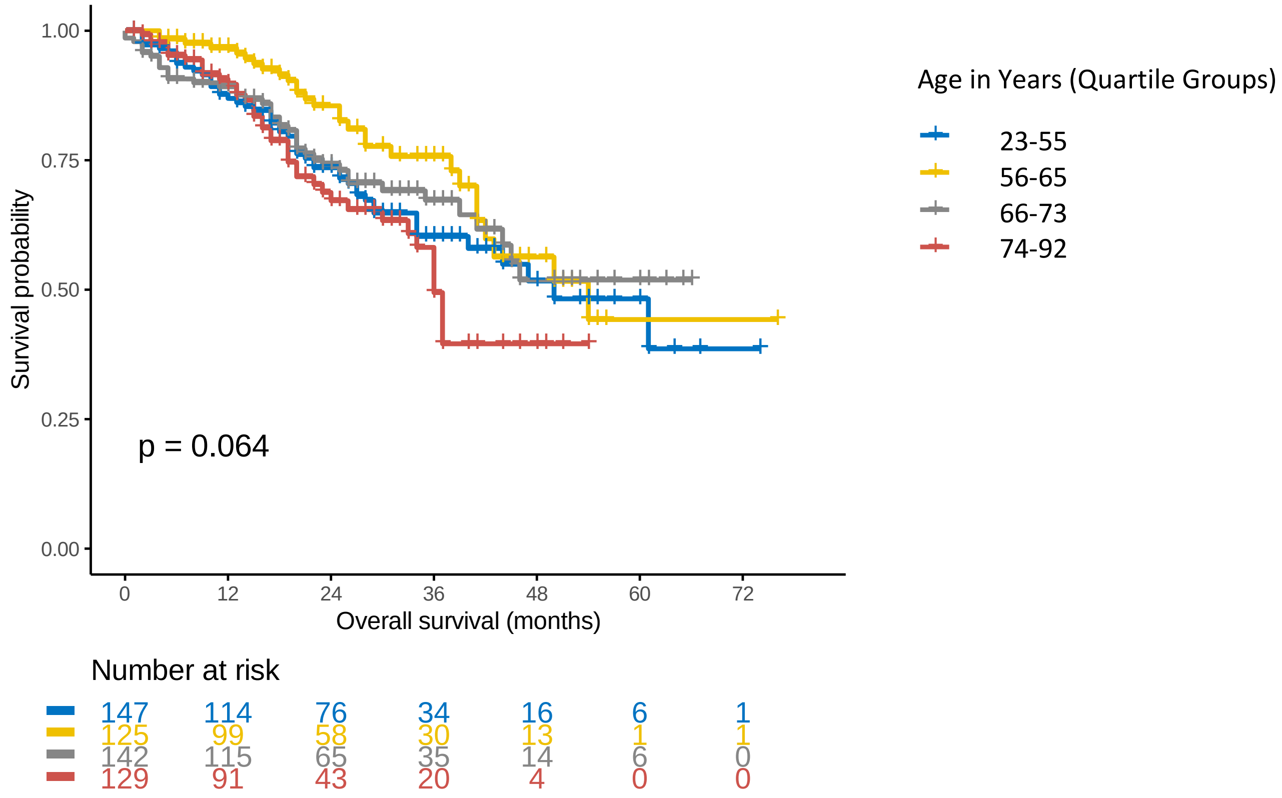

Supplement: Supplementary Figure 1 [file mmc6.pdf]
