# Supplementary material for: UK multicentre real-world data of the use of cyclin-dependent kinase 4/6 inhibitors in metastatic breast cancer
Source: ESMO Real World Data Digit Oncol. 2024 Aug 20;5:100064. doi: 10.1016/j.esmorw.2024.100064 (PMC12836663; doi:10.1016/j.esmorw.2024.100064)

Supplementary Figure 2. (A) Kaplan-Meier Analysis of progression free survival (PFS) rates for all patients in the cohort receiving CDK4/6 inhibitors as a second line therapy or beyond for ER+/HER2- MBC (B) Kaplan-Meier Analysis of overall survival (OS) for all patients in the cohort receiving CDK4/6 inhibitors as second line therapy or beyond therapy for ER+/HER2- MBC

4A

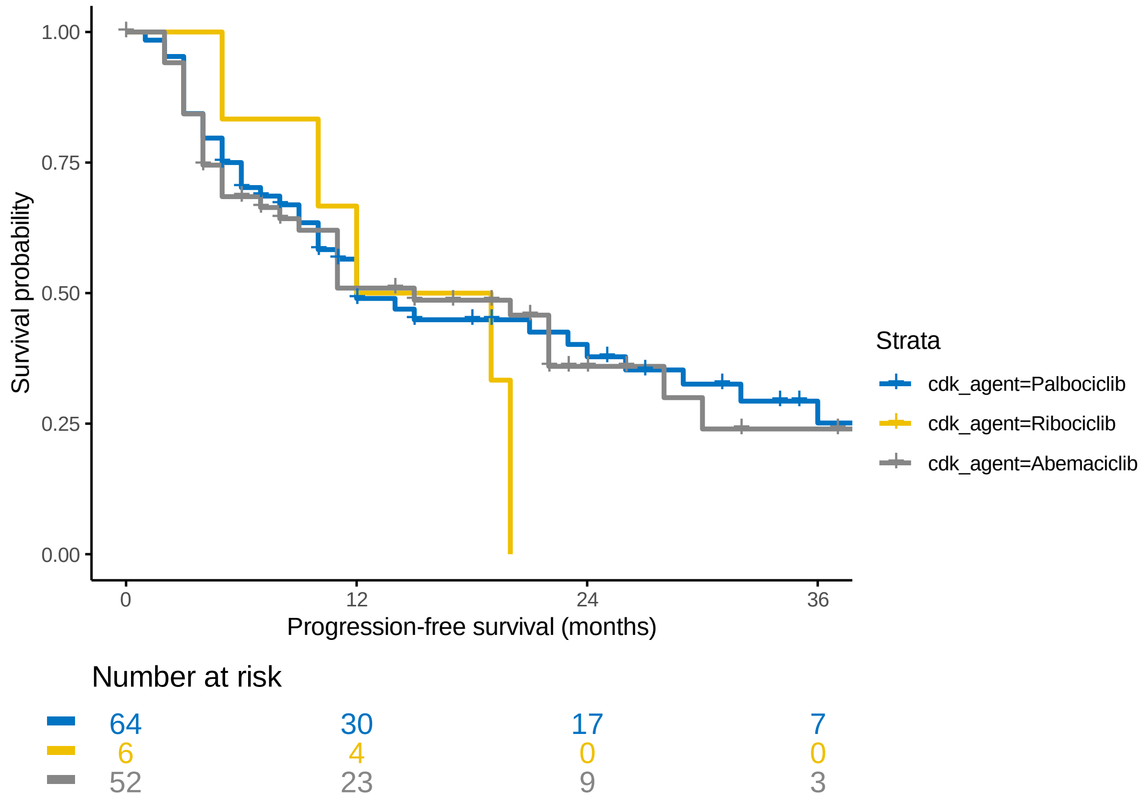

4B

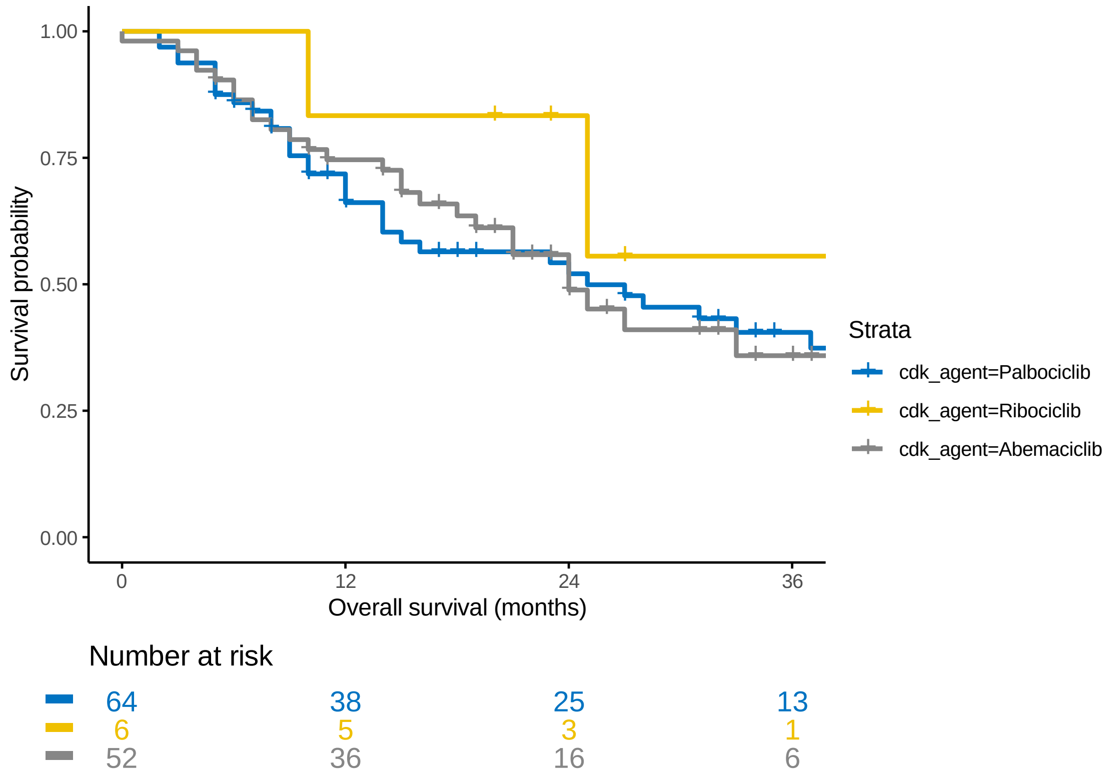

Supplement: Supplementary Figure 2 [file mmc7.pdf]
